# 免疫检查点抑制剂一线治疗晚期NSCLC的现状与展望

**DOI:** 10.3779/j.issn.1009-3419.2017.06.10

**Published:** 2017-06-20

**Authors:** 海洋 王

**Affiliations:** 310053 杭州，浙江省肿瘤医院 Department of Medical Oncology, Zhejiang Provincial Cancer Hospital, Hangzhou 310053, China

**Keywords:** PD-1/PD-L1抗体, 肺肿瘤, 一线治疗, PD-1/PD-L1 inhibitors, Lung neoplasms, First-line treatment

## Abstract

随着程序性凋亡因子1受体（programmed death-1, PD-1）及其配体（PD-L1）抑制剂单药治疗在晚期非小细胞肺癌（non-small cell lung cancer, NSCLC）的二线和一线治疗中相继取得突破性进展，晚期NSCLC的诊治策略正在逐渐发生演变和优化。免疫联合治疗扩大受益人群、提高疗效，目前已经在一线治疗领域取得初步结果，有多项Ⅲ期随机对照研究正在进行中。本文将对免疫检查点抑制剂在晚期NSCLC一线治疗中的现状和前景进行综述。

## Abstract (en)

With the breakthroughs achieved of programmed death-1 (PD-1)/PD-L1 inhibitors monotherapy as first-line and second-line treatment in advanced non-small cell lung cancer (NSCLC), the treatment strategy is gradually evolving and optimizing. Immune combination therapy expands the benefit population and improves the curative effect. A series of randomized phase Ⅲ trials are ongoing. In this review, we discuss the prospect and current situation of immune checkpoint inhibitors in first-line treatment in advanced NSCLC patients.

程序性凋亡因子1受体（programmed death-1, PD-1）及其配体（PD-L1）是一对负性免疫共刺激分子，肿瘤细胞表达的PD-L1，可与肿瘤浸润淋巴细胞（tumor infiltrating lymphocytes, TILs）表面的PD-1分子结合，抑制淋巴细胞的功能及细胞因子的释放，并诱导淋巴细胞凋亡，从而抵抗淋巴细胞的杀伤作用，最终导致肿瘤发生免疫逃逸^[1]^。PD-1/PD-L1抗体可阻断PD-L1/PD-1通路，从而使被抑制的效应T细胞（effector T cell, TE）功能恢复。

在过去的2年中，已经有3个PD-1/PD-L1抗体进入晚期非小细胞肺癌（non-small cell lung cancer, NSCLC）的二线治疗。Nivolumab、Pembrolizumab、Atezolizumab分别凭借CheckMate017、CheckMate057、KEYNOTE010及OAK研究获得晚期NSCLC二线治疗的适应症^[2-5]^。这些大型、Ⅲ期随机对照研究在含铂两药方案化疗失败的晚期NSCLC中比较PD-1/PD-l1抗体与多西他赛的疗效，结果表明Nivolumab、Pembrolizumab、Atezolizumab在缓解率（overall response rate, ORR）、无进展生存时间（progression-free survival, PFS）或/及总生存（overall survival, OS）上明显好于标准二线化疗多西他赛，由此奠定了PD-1/PD-L1抗体在晚期NSCLC二线治疗中的地位。

自20世纪70年代起，含铂化疗一直是晚期NSCLC的标准一线治疗。尽管化疗的疗效有限且不良反应明显，囿于治疗手段的匮乏，对于野生型患者而言，一线治疗并未获得突破性进展。随着PD-1/PD-L1抗体在晚期NSCLC的二线治疗中得到认可，应用于一线治疗的相关研究正在火热进行中。目前已有两项Ⅲ期随机对照研究公布了结果^[6, 7]^，多项大型Ⅲ期临床研究仍在入组中。本文就PD-1/PD-L1抗体在晚期NSCLC一线治疗中的现状、未来的发展方向进行全面阐述。

## 1 单药的一线治疗

多项Ⅲ期随机对照研究的结果显示，在未经选择的人群，PD-1/PD-L1抗体二线治疗晚期NSCLC的ORR为14.0%-20.0%，PFS为3.5个月-4.0个月，OS为9.2个月-13.8个月^[2-5]^。这样的疗效与标准二线治疗药物多西他赛7%左右的近期ORR相比^[2-5]^，均表现出优越性。但是，在晚期NSCLC的一线治疗中，含铂两药方案的ORR达25%-35%^[8-10]^；如果想超越这个疗效，势必要用生物标记物来富集治疗有效人群。到目前为止，主要的策略是用PD-L1的表达来筛选患者。为此，一些Ⅰ期-Ⅱ期临床研究进行了相应地探索。在KEYNOTE 001研究中^[11]^，Pembrolizumab单药治疗晚期NSCLC的ORR为24.8%；但在PD-L1表达≥50%的初诊人群（*n*=27），ORR上升到58%，24个月OS达到61%。在CheckMate 012研究中^[12]^，52例晚期NSCLC患者一线接受Nivolumab单药治疗，ORR为23%，mPFS为3.6个月；在PD-L1高表达（PD-L1≥50%）人群（*n*=12），ORR高达50%，PFS为8.3个月，1年生存率为83%。2016年美国临床肿瘤学会（American Society of Clinical Oncology, ASCO）会议上报道了抗PD-L1抗体Durvalumab（MEDI4736）治疗初诊晚期NSCLC患者的安全性和临床活性^[13]^，共入组59例患者，其中PD-L1高表达患者49例，Durvalumab治疗剂量10 mg/kg q2w，全组患者的ORR为27%，PD-L1高表达患者的ORR为29%，PD-L1低表达/阴性患者的ORR为11%；10%患者出现了≥3级的药物相关不良事件，安全性可控。2016年世界肺癌大会（World Conference on Lung Cancer, WCLC）会议报告了一项Ⅱ期研究（BIRCH研究）^[14]^，评价Atezolizumab治疗PD-L1表达的未经化疗的晚期NSCLC，入组138例PD-L1表达（TC2/3和或IC2/3）阳性患者，在TC或者IC2/3的患者，ORR为25%，mPFS为7.3个月，mOS为23.5个月；在TC3或者IC3的患者，ORR为34%，mPFS为7.3个月，mOS为26.9个月。评价Atezolizumab对比化疗一线治疗NSCLC的Ⅲ期临床研究正在进行中。

最引入关注的是两项Ⅲ期随机对照KEYNOTE-024和CheckMate026研究^[5, 6]^，分别在PD-L1阳性人群中比较一线含铂化疗方案与Pembrolizumab或Nivolumab。KEYNOTE-024研究将目标人群定位在PD-L1表达≥50%的晚期NSCLC^[5]^，入组305例患者。所有受试者被随机分配至Pembrolizumab组（200 mg/3 wk，静脉滴注）和含铂化疗组。研究发现，Pembrolizumab组的中位PFS为10.3个月，明显长于化疗组的6.0个月（HR=0.5, *P* < 0.001）；ORR（45% *vs* 28%, *P*=0.001）和OS（中位OS两组均为达到，HR=0.6，*P*=0.005）均显著优于化疗组。Pembrolizumab治疗组3级-4级不良反应的发生率明显低于多西他赛（26% *vs* 51%）。同时，Pembrolizumab可显著改善患者的健康相关生活质量（health-related quality of life, HRQOL）^[15]^，可使患者的症状和功能得到改善或维持在较高水平，显著延长至疾病恶化时间。基于KEYNOTE-024研究的结果，FDA已批准Pembrolizumab作为PD-L1表达TPS≥50%的晚期NSCLC新的标准一线治疗。

与此形成鲜明对照的是CheckMate 026研究^[6]^，其设计与KEYNOTE-024研究相类似，主要不同的是入组人群，以PD-L1表达≥1%的Ⅳ期初诊NSCLC作为研究对象，以PD-L1表达≥5%的患者作为评价疗效的目标人群，共541例患者进入分析，结果Nivolumab未达到预设的主要研究终点，PFS对比单纯化疗未显示出优势（4.2个月*vs* 5.9个月，*P*=0.25），两组的ORR（26% *vs* 33.5%）和OS（14.4个月*vs* 13.2个月，HR=1.02）亦无统计学差异。

在PD-L1表达TPS≥50%的晚期NSCLC中，Pembrolizumab一线治疗的疗效已经得到确认。一些关键问题随之而来。首先是关于PD-L1高表达的cut-off值问题，TPS≥50%已经得到认可，TPS≥25%是否可行，目前已有针对PD-L1表达TPS≥25%人群的相关研究。第二，关于PD-L1的检测问题，如何对不同的检测试剂、检测平台、检测对象进行标准化评估。蓝印计划的结果显示^[16]^，检测PD-L1表达的常用三种抗体 22C3、28-8和SP263，在检测率上有高度的一致性，是否这3种抗体及检测平台的结果可以互通^[16]^。第三，由于PD-L1表达≥50%的人群只占约30%的肺癌患者，是否在占比高达70%的PD-L1低表达或者不表达人群，PD-1/PD-L1抗体在一线治疗将就此失去机会。答案显然是否定的，免疫联合治疗为这类患者带来了希望，其中主要是PD-1/PD-L1抗体与化疗或者是CTAL4抗体的联合。

## 2 PD-1/PD-L1抗体联合化疗

细胞毒药物具有多方面的正向免疫调节作用，降低免疫抑制性细胞的数量和活性，增加肿瘤抗原递呈，激活和诱导树突状细胞成熟，增加效应T细胞功能，诱导肿瘤细胞PD-L1表达等等^[16-19]^。前期研究表明化疗联合PD-1/PD-L1抗体具有协同抗肿瘤作用^[17-19]^。KEYNOTE-021 C队列研究采用Pembrolizumab 2 mg/kg或10 mg/kg q3w联合卡铂/培美曲塞治疗晚期、初诊非鳞NSCLC^[20]^，入组24例患者，ORR为71%，PFS为10.2个月，中位OS未达到；1例患者因3级表皮坏死导致治疗中断，无预期外毒副反应，无治疗相关死亡。KEYNOTE-021 G队列研究是一项Ⅱ期的随机对照研究^[20]^，旨在未经化疗的晚期非鳞NSCLC患者中比较Pembrolizumab联合卡铂+培美曲赛与单纯化疗，筛选了219例患者，123例患者可评价疗效，Pembrolizumab联合化疗组相比单纯化疗组明显提高了ORR（55% *vs* 29%, *P*=0.001, 6），显著延长了PFS（13.0个月*vs* 8.9个月，*P*=0.01，HR=0.53），两组的OS无明显差异。在PD-L1≥50%的亚组人群，联合治疗组的ORR高达80%，显著高于化疗组的35%。联合治疗组≥3度的治疗相关不良反应发生率高于化疗组（39% *vs* 26%），但总体不良反应可耐受，不良反应谱可管理。这是首个评价PD-1/PD-L1抗体联合化疗对比单纯化疗的Ib期随机对照研究。目前，Pembrolizumab联合化疗已获美国食品和药物管理局（Food and Drug Administration, FDA）突破性治疗药物资格。其他PD-1/PD-L1单抗，如Nivolumab和Atezolizumab均进行了与化疗联合的相关研究。前期报道的Ⅰ期研究显示Nivolumab和Atezolizumab联合含铂方案化疗一线治疗晚期NSCLC的缓解率分别达47%和64%^[7, 21]^。这些Ⅰ期研究的结果显示PD-1/PD-L1抗体联合含铂方案化疗的近期疗效具有可比性，在晚期NSCLC的一线治疗中有望超越单纯化疗；是否能如愿斩获一线治疗的适应症，仍需大样本的Ⅲ期随机对照研究。现阶段多个PD-1/PD-L1抗体均有多项相关Ⅲ期随机临床试验正在入组中，以评估这些联合模式在晚期NSCLC一线治疗中的作用。

## 3 PD-1/PD-L1抗体联合CTLA-4抗体

除了联合化疗，另一种引入关注的组合是PD-1/PD-L1抗体联合靶向细胞毒T淋巴细胞相关抗原4（cytotoxic T lymphocyte-associated antigen-4, CTLA-4）抗体。CTLA-4通路对于淋巴结部位的T细胞活化起作用，CTLA-4抗体能使T细胞活化增殖能力变强，并且使T细胞分化为效应T细胞，如CTL。CTLA-4和PD-1这两个抑制剂会在不同的环节和不同阶段对T细胞功能进行加强。临床前研究提示两者联合可放大checkpoint阻断作用，在免疫微环境中，充分恢复T细胞杀伤肿瘤细胞的效能^[1, 22]^。CheckMate 012研究是一项多中心Ib期临床研究^[23]^，分别评价Nivolumab单药、Nivolumab联合Ipilimumab的不同给药方式治疗未经化疗的晚期NSCLC，联合治疗组77例患者可评价疗效，总体ORR为43%，mPFS为8.0个月，2年生存率为44%，mOS为21.8个月。Nivolumab联合Ipilimumab的疗效在PD-L1≥1%人群，ORR为57%，mPFS达12.7个月；在PD-L1≥50%亚组，ORR高达92%，mPFS为13.6个月。Nivolumab联合Ipilimumab治疗的不良反应可耐受，不良反应谱同既往报道，NIVO 3 q2w+IPI 1 q12w治疗组（*n*=38）的不良反应发生率稍高于NIVO 3 q2w+IPI 1 q6w治疗组（*n*=39），3级-4级毒性的发生率分别为42%和31%，延长随访后未发现新的安全性问题或治疗相关死亡。CheckMate 012研究结果证明Nivolumab联合Ipilimumab在PD-L1≥1%的人群显示出良好疗效，其疗效随着肿瘤细胞PD-L1表达的增高而增强。此结果鼓励在晚期NSCLC的一线治疗中开展Nivolumab联合Ipilimumab的进一步研究。目前Ⅲ期随机对照研究CheckMate 227（NCT02477826）已经在进行中。

PD-L1抗体（Durvalumab）联合CTLA-4抗体（Tremelimumab）的Ib期研究在102例初诊的晚期NSCLC中进行^[24]^，严重不良事件（serious adverse event, SAE）的发生率达36%，约28%的患者中断治疗。基于前期研究的安全性及临床资料，在剂量扩增研究中，推荐治疗剂量Durvalumab 20 mg/kg加Tremelimumab 1 mg/kg，每4周1次；在63例可评价疗效的患者中，ORR为23%。Durvalumab联合Tremelimumab对比含铂两药化疗一线治疗初诊晚期NSCLC的Ⅲ期随机对照MYSTIC研究（NCT02453282）和NEPTUNE研究（NCT02542293）正在入组中。

## 4 PD-1/PD-L1抗体联合其他药物

肿瘤血管生成与肿瘤的免疫抑制相互促进，相互影响。临床前研究显示VEGF通过不同的机制在免疫抑制调节中发挥着重要的作用，同时免疫抑制细胞对肿瘤血管生成具有促进作用，因此抗血管生成治疗联合免疫治疗具有协同效应^[25]^。临床数据显示：贝伐珠单抗联合干扰素、CTLA-4抑制剂取得了较好的临床疗效^[27]^，且耐受性良好。目前多个贝伐珠单抗联合免疫治疗（Checkpoint抑制剂及疫苗）的研究正在开展，尤其是贝伐珠单抗联合化疗及Atezolizumab的随机对照研究已进入Ⅲ期临床，结果值得期待。

靶向药物是驱动基因阳性患者的主要治疗手段，与PD-1/PD-L1抗体的强强联合是否能达到增效作用。TATTON研究评价Osimertinib联合Durvalumab治疗EGFR突变的晚期NSCLC^[26]^，A组23例为EGFR-TKI治疗失败患者，B组11例为初诊患者，结果发现联合治疗在A组有6例（26%）患者出现间质性肺炎，2例为3级/4级；B组有7（64%）例患者出现间质性肺炎，3级/4级3例。一项Ⅰ期、开放性、多中心研究（NCT02040064）评价Tremelimumab联合吉非替尼在EGFR突变的NSCLC患者中安全性、耐受性和临床疗效，针对吉非替尼/厄洛替尼治疗失败的Ⅳ期NSCLC患者，共入组20例，至少接受1个剂量Tremelimumab治疗，17例患者可评估疗效及安全性，无完全缓解（complete response, CR）和部分缓解（partial response, PR）患者，DCR 62%，联合用药的不良反应谱与单药一致^[27]^。鉴于PD-1/PD-L1抗体在EGFR突变患者中的疗效偏低^[28]^，结合一些小样本的初步研究结果，PD-1/PD-L抗体联合EGFR TKIs可能有较高的间质性肺炎发生率；该联合治疗模式受到一定质疑，现阶段正在进行的主要是Ⅰ期探索性研究。

CheckMate 012研究中，21例*EGFR*突变的患者经厄洛替尼联合Nivolumab治疗后，19%的患者出现了3级毒副反应，但没有出现4级毒副反应；其ORR为19%，51%的患者PFS达到了24周，64%的患者OS达到了18周，其研究终点尚未达到^[29]^。在另一项正在进行的Ib期研究中，28例患者接受Atezolizumab联合厄洛替尼治疗，没有患者因存在不可耐受的毒性而停药，重要的是并未观察到间质性肺炎；然而在39%的患者中观察到了3级-4级的毒副反应，包括谷氨酸氨基转移酶（alanine aminotransferase, ALT）升高（7%）、发热（7%）和皮疹（7%）^[30]^。

T细胞的活化受抑制性和刺激性受体双重调节作用，如PD-1、CTLA4抑制T细胞的活化, 刺激性分子激活增强T细胞受体的信号通路。OX40促进抗原依赖性效应T细胞的活化并调节抑制T细胞。OX40激动剂具有双重作用机制，刺激效应T细胞、调节抑制性T细胞。OX40激动剂MOXR0916是一种人工合成的IgG1抗体，临床前研究证明OX40激动剂和PD-L1抑制剂联合具有互补效应。在2016年ASCO会议上报道了OX40激动剂MOXR0916联合PD-L1抑制剂Atezolizumab治疗晚期实体肿瘤患者的剂量递增Ib期研究，结果显示饱和剂量的MOXR0916在与Atezolizumab联合使用时耐受良好，未达到剂量限制性毒性^[31]^。

## 5 小结

基于KEYNOTE-024研究的结果，PD-L1≥50%的晚期、野生型NSCLC的一线治疗模式已经发生改变，推荐Pembrolizumab单药作为一线治疗；相信不久的将来，会有多个PD-1/PD-L1抗体进入可供选择的行列。对于PD-L1低表达或者不表达的人群，主要的解决之道是联合治疗。目前报道的PD-1/PD-L1抗体联合其他药物的临床研究均为Ⅰ期/Ⅱ期，普遍存在样本量小、随访期短、入组人群可能有选择偏倚等缺陷。基于前期研究的良好结果，多种模式的免疫联合治疗在进一步探索中。在晚期NSCLC的一线治疗中，众多大型、Ⅲ期随机对照的临床研究正在如火如荼进行；如CheckMate 227研究、KEYNOTE-189研究、IMpower130研究、IMpower131研究、MYSTIC研究、NEPTUNE研究等等^[8]^。这些研究聚焦在晚期NSCLC的初诊患者，选择不同的PD-L1表达人群，将回答PD-1/PD-L1抗体联合化疗、CTLA4抗体或者抗血管生存药物等作为一线治疗的可行性、适用人群及不同联合模式之间的差异性。相比PD-1/PD-L1抗体的单药治疗，免疫联合治疗扩大了受益人群，提高了疗效，是未来的发展方向。免疫联合治疗仍有许多问题亟待解决：如最佳的联合搭档；最合适的给药方式；不良反应的管理；生物标记物的筛选；药物经济学等等。可以预见，Pembrolizumab一线治疗PD-L1高表达人群仅仅是即将来临的新的免疫治疗策略的前奏，晚期NSCLC一线治疗的格局将会发生革命性的变化。

## References

[1] Pardoll DM (2012). The blockade of immune checkpoints in cancer immunotherapy. Nat Rev Cancer.

[2] Brahmer J, Reckamp KL, Baas P (2015). Nivolumab versus docetaxel in advanced squamous-cell on-small-cell lung cancer. N Engl J Med.

[3] Borghaei H, Paz-Ares L, Horn L (2015). Nivolumab versus docetaxel in advanced nonsquamous non-small-cell lung cancer. N Engl J Med.

[4] Herbst RS, Baas P, Kim DW (2016). Pembrolizumab versus docetaxel for previously treated, PD-L1-positive, advanced non-small-cell lung cancer (KEYNOTE-010): a randomised controlled trial. Lancet.

[5] Fehrenbacher L, Spira A, Ballinger M (2016). Atezolizumab versus docetaxel for patients with previously treated non-small-cell lung cancer (POPLAR): A multicentre, open-label, phase 2 randomised controlled trial. Lancet.

[6] Reck M, Rodriguez-Abreu D, Robinson AG (2016). Pembrolizumab versus chemotherapy for PD-L1-positive non-small-cell lung cancer. N Engl J Med.

[7] Rizvi NA, Hellmann MD, Brahmer JR (2016). Nivolumab in combination with platinum-based doublet chemotherapy for first-linetreatment of advanced non-small-cell lung cancer. J Clin Oncol.

[8] Schiller JH, Harrington D, Belani CP (2002). Comparison of four chemotherapy regimens for advanced non-small-cell lung cancer. N Engl J Med.

[9] Scagliotti GV, Parikh P, von Pawel J (2008). Phase Ⅲ study comparing cisplatin plus gemcitabine with cisplatin plus pemetrexed in chemotherapy naive patients with advanced-stage non-small-cell lung cancer. J Clin Oncol.

[10] Sandler A, Gray R, Perry MC (2006). Paclitaxel-carboplatin alone or with bevacizumab for non-small-cell lung cancer. N Engl J Med.

[11] Garon EB, Rizvi NA, Hui R (2015). EYNOTE-001 Investigators. Pembrolizumab for the treatment of non-small-cell lung cancer. N Engl J Med.

[12] Gettinger S, Rizvi NA, Chow LQ (2016). Nivolumab monotherapy for first-line treatment of advanced non-small-cell lung cancer. J Clin oncol.

[13] Antonia SJ, Kim SW, Alexander I (2016). Safety and clinical activity of durvalumab (MEDI4736), an anti-PD-L1 antibody, in treatment-na?ve patients with advanced non?small-cell lung cancer. Proc Am Soc Clin Oncol.

[14] Wakelee H, Patel JD, Heist R (2016). ORAL01.04: Phase Ⅱ trialof atezolizumab for patients with PD-L1-selected advanced NSCLC (BIRCH): updated efficacy and exploratory biomarker results: topic: medical oncology. J Thorac Oncol.

[15] Reck M, Rodríguez-Abreu D, Robinson AG (2016). Pembrolizumab versus chemotherapy for PD-L1-positive non-small-cell lung cancer. N Engl J Med.

[16] Hirsch FR, McElhinny A, Stanforth D (2017). PD-L1 immunohistochemistry assays for lung cancer: results from phase 1 of the blueprint PD-L1 IHC assay comparison project. J Thorac Oncol.

[17] Zitvogel L, Galluzzi L, Smyth MJ (2013). Mechanism of action of conventional and targeted anticancer therapies: reinstating immuneosurveillance. Immunity.

[18] Galluzzi L, Buque A, Kepp O (2015). Immunological effects of conventional chemotherapy and targeted anticancer agents. Cancer Cell.

[19] Apetoh L, Ladoire S, Coukos G (2015). Combining immunotherapy and anticancer agents: the right path to achieve cancer cure?. Ann Oncol.

[20] Langer CJ, Gadgeel SM, Borghaei H (2016). Carboplatin and pemetrexed with or without pembrolizumab for advanced, nonsquamous non-small-cell lung cancer: a randomised, phase 2 cohort of the open-label KEYNOTE-021 study. Lancet Oncol.

[21] Camidge DR, Liu SV, Powderly JD (2015). Atezolizumab (MPDL3280A) combined with platinum-based chemotherapy in non-small cell lung cancer (NSCLC): a phase 1b study. J Thorac Oncol.

[22] Curran MA, Montalvo W, Yagita H (2010). PD-1 and CTLA-4combination blockade expands infiltrating T cells and reduces regulatory T and myeloid cells within B16 melanoma tumors. Proc Natl Acad Sci USA.

[23] Hellmann MD, Rizvi NA, Goldman JW (2017). Nivolumab plus ipilimumab as first-line treatment for advanced non-small-cell lung cancer (CheckMate 012): results of an open-label, phase 1, multicohort study. Lancet Oncol.

[24] Antonia S, Goldberg SB, Balmanoukian A (2016). Safety and antitumouractivity of durvalumab plus tremelimumab in non-small cell lung cancer: a multicenter phase 1b study. Lancet Oncol.

[25] Mahoney KM, Rennert PD, Freeman GJ (2015). Combination cancer immunotherapy and new immunomodulatory targets. Nat Rev Drug Discov.

[26] Ahn MJ, Yang J, Yu H (2016). Osimertinib combined with durvalumab in *EGFR*-mutant non-small cell lung cancer: results from the TATTON phase Ib trial. J Thorac Oncol.

[27] Planchard D, Barlesi F, Gomez-Roca C (2016). Phase Ⅰ, safety, tolerability and preliminary efficacy study of tremelimumab (Trem) in combination with gefitinib (Gef) in *EGFR*-mutant (*EGFR*-mut) NSCLC (GEFTREM). Ann Oncol.

[28] LEE CK, Man J, Lord S (2017). Brief report: checkpoint inhibitors in metastatic *EGFR*-mutated non-small cell lung cancer-a *meta*-analysis. J Thorac Oncol.

[29] Antonia SJ, Rizvi NA, Chow LQ (2014). Nivolumab (anti-PD-1; Bms-936558, Ono-4538) in combination with platinum-based doublet chemotherapy (Pt-DC) or erlotinib in advanced non-small cell lung cancer (NSCLC). J Thorac Oncol.

[30] Ma BBY, Rudin CM, Cervantes A (2016). Preliminary safety and clinical activity of erlotinib plus atezolizumab from a phase Ib study in advanced NSCLC. Ann Oncol.

[31] Infante JR, Hansen AR, Pishvaian MJ (2016). A phase Ib dose escalation study of the OX40 agonist MOXR0916 and the PD-L1 inhibitor atezolizumab in patients with advanced solid tumors. Proc Am Soc Clin Oncol.

